# The E46K mutation modulates α-synuclein prion replication in transgenic mice

**DOI:** 10.1371/journal.ppat.1010956

**Published:** 2022-12-01

**Authors:** Sara A. M. Holec, Jisoo Lee, Abby Oehler, Lyn Batia, Aryanna Wiggins-Gamble, Jeffrey Lau, Felicia K. Ooi, Gregory E. Merz, Man Wang, Daniel A. Mordes, Steven H. Olson, Amanda L. Woerman

**Affiliations:** 1 Department of Biology and Institute for Applied Life Sciences, University of Massachusetts Amherst; Amherst, Massachusetts, United States of America; 2 Institute for Neurodegenerative Diseases, Weill Institute for Neurosciences, University of California, San Francisco; San Francisco, California, United States of America; 3 Department of Neurology, University of California, San Francisco; San Francisco, California, United States of America; 4 Department of Pathology, University of California, San Francisco; San Francisco, California, United States of America; University of Edinburgh, UNITED KINGDOM

## Abstract

In multiple system atrophy (MSA), the α-synuclein protein misfolds into a self-templating prion conformation that spreads throughout the brain, leading to progressive neurodegeneration. While the E46K mutation in α-synuclein causes familial Parkinson’s disease (PD), we previously discovered that this mutation blocks *in vitro* propagation of MSA prions. Recent studies by others indicate that α-synuclein adopts a misfolded conformation in MSA in which a Greek key motif is stabilized by an intramolecular salt bridge between residues E46 and K80. Hypothesizing that the E46K mutation impedes salt bridge formation and, therefore, exerts a selective pressure that can modulate α-synuclein strain propagation, we asked whether three distinct α-synuclein prion strains could propagate in TgM47^+/-^ mice, which express human α-synuclein with the E46K mutation. Following intracranial injection of these strains, TgM47^+/-^ mice were resistant to MSA prion transmission, whereas recombinant E46K preformed fibrils (PFFs) transmitted neurological disease to mice and induced the formation of phosphorylated α-synuclein neuropathology. In contrast, heterotypic seeding following wild-type (WT) PFF–inoculation resulted in preclinical α-synuclein prion propagation. Moreover, when we inoculated TgM20^+/-^ mice, which express WT human α-synuclein, with E46K PFFs, we observed delayed transmission kinetics with an incomplete attack rate. These findings suggest that the E46K mutation constrains the number of α-synuclein prion conformations that can propagate in TgM47^+/-^ mice, expanding our understanding of the selective pressures that impact α-synuclein prion replication.

## Author summary

The underlying cause of disease in a group of movement disorders called syncleinopathies is the misfolding of the protein α-synuclein into a shape that self-templates to spread disease. The type of synucleinopathy that a patient develops depends on the shape α-synuclein adopts during misfolding. For example, α-synuclein misfolds into one shape in patients with Parkinson’s disease (PD) and into another in patients with multiple system atrophy (MSA). These distinct forms are referred to as strains. Understanding α-synuclein strain biology, and the factors that impact strain formation and spread, is imperative for developing successful diagnostics and therapeutics. In this study, we investigated the effect of the E46K mutation in α-synuclein, which causes PD, on α-synuclein strain biology to determine how the patient’s genetic background can impact disease. Our results showed that this particular mutation modulates α-synuclein spread; while mice expressing E46K α-synuclein were resistant to MSA prion replication, synthetic E46K fibrils induced neurological disease in the mice. In contrast, heterotypic seeding of wild-type synthetic fibrils yielded inefficient propagation that could not induce neurological signs in the mice. These results indicate that patient genotype can greatly impact α-synuclein strain formation and, therefore, clinical presentation in synucleinopathy patients.

## Introduction

Synucleinopathies are a group of neurodegenerative diseases caused by the misfolding and self-templating of the α-synuclein protein. In one group of diseases, α-synuclein misfolds and aggregates into neuronal Lewy bodies and Lewy neurites [1]. This group, collectively referred to as Lewy body diseases (LBDs), includes Parkinson’s disease (PD), PD with dementia (PDD), and dementia with Lewy bodies (DLB). In contrast, in patients with multiple system atrophy (MSA), α-synuclein misfolds into glial and neuronal cytoplasmic inclusions. While MSA and LBD patients can present with overlapping motor deficits, MSA patients are typically diagnosed earlier in life (50–60 years old rather than >70 years old) and experience a more rapid disease progression compared to LBD patients [2–4]. However, while there are eleven known familial LBD mutations in the gene encoding α-synuclein, *SNCA*, no mutations have been identified in MSA patients [5–16].

To investigate the role of α-synuclein misfolding and self-templating in synucleinopathies, we previously inoculated TgM83^+/-^ mice, which express human SNCA*A53T [17], with homogenates prepared from brain samples of deceased MSA patients [18,19]. Unexpectedly, while the MSA patient samples induced neurological disease ~120 days postinoculation (dpi), none of the PD, DLB, or PDD patient samples tested transmitted disease to the mice. More recently, Thomzig *et al*. found that brain tissue from two deceased PD patients induced subclinical neuropathological changes in the brain, absent the onset of neurological signs [20]. In parallel, we used a HEK293T cell assay expressing α-syn140*A53T–YFP to propagate α-synuclein prions isolated from MSA patient samples but not those from the PD or DLB patients [21]. These findings support our hypothesis that MSA is caused by α-synuclein misfolding into a distinct conformation, or strain, compared to the α-synuclein fibrils present in LBD patients.

Being unable to transmit DLB prions in cultured cells or Tg mice, we built a panel of α-syn–YFP cell lines expressing *SNCA* mutations and tested the ability of MSA prions to replicate using mutant protein [22]. We found that MSA prions can propagate using wild-type (WT), A30P, and A53T α-synuclein, but the presence of the PD-causing E46K mutation prevented MSA prion replication *in vitro*. That a single point mutation in α-synuclein interferes with prion propagation is not surprising; the *Canidae* family was found to be resistant to PrP prion disease due to the presence of a single residue polymorphism at position 163 in the PrP protein [23]. Additionally, the Q171R mutation in sheep and the E219K mutation in humans both confer resistance to prion disease [24–26].

Several lines of research have focused on understanding why the E46K mutation exhibits a unique effect on α-synuclein pathogenesis. Testing the ability of WT α-synuclein preformed fibrils (PFFs) to induce neuropathological inclusions in TgM47 mice, which express SNCA*E46K [27], Sacino *et al*. reported inefficient transmission with limited inclusions around the injection site at 4 months postinoculation (mpi) [28]. Notably, similar studies in the TgM83 animals resulted in widespread neuronal inclusions throughout the brain. In 2016, Tuttle *et al*. used solid-state nuclear magnetic resonance to resolve the structure of WT α-synuclein fibrils and reported that a salt bridge between residues E46 and K80 stabilizes a Greek key motif within the fibril structure [29]. Subsequent cryo-electron microscopy (cryo-EM) studies have identified the E46/K80 salt bridge in WT recombinant fibrils as well as fibrils isolated from MSA patient samples [30–34]. Consistent with the hypothesis that the E46K mutation disrupts this interaction, the lack of the salt bridge in two cryo-EM structures resolved using E46K fibrils results in conformations that vary substantially from those reported for WT fibrils [35,36]. Moreover, Long *et al*. showed that WT fibrils containing the E46/K80 salt bridge are unable to propagate E46K α-synuclein prions, but E46K fibrils can template using WT protein as a substrate [37]. It is important to note that the cryo-EM structures reported using either WT or mutant PFFs vary substantially from the conformations resolved using MSA patient samples (reviewed in [38]). These conformations are also structurally distinct from the recently resolved α-synuclein fibrils isolated from LBD patient samples, which show that K80 forms a salt bridge with E35, rather than E46, in LBD patients [39]. Altogether, a growing body of literature suggests that the E46K mutation is likely to exert selective pressures on α-synuclein prion propagation different from the effects of other familial *SNCA* mutations.

To understand the effect of the E46K mutation on α-synuclein prion strain propagation, we investigated the ability of three different α-synuclein prion sources to replicate in TgM47^+/-^ mice. Consistent with our cell assay data, the E46K mutation inhibited MSA transmission to the mice over a 475-day incubation period. Conversely, inoculations using E46K PFFs transmitted disease to the animals; however, a delayed incubation period suggests that the kinetics of E46K α-synuclein self-templating are slowed compared to the well-studied A53T mutation. Distinct from both of these results, we observed that heterotypic seeding in which TgM47^+/-^ mice were inoculated with WT PFFs resulted in preclinical α-synuclein prion propagation. Finally, we reversed this experiment and tested inoculation of E46K PFFs into the TgM20^+/-^ mouse model, which expresses WT human α-synuclein [17], and observed disease transmission with an incomplete attack rate. Our findings not only indicate that prion propagation using E46K protein as substrate is limited to a subset of α-synuclein conformations, but they also contribute to our understanding of the selective pressures that impact α-synuclein prion replication.

## Results

### The PD-causing E46K mutation in α-synuclein inhibits MSA prion transmission

Previously, we used a panel of α-syn–YFP cell lines to demonstrate that the E46K mutation, which causes PD and DLB [7], interferes with *in vitro* replication of MSA prions [22]. To determine if our findings in cultured cells are predictive of *in vivo* α-synuclein strain biology, we investigated whether MSA prions can transmit disease to transgenic (Tg) mice expressing the same mutation. The TgM47 mouse model uses the *Prnp* promoter to drive expression of human α-synuclein with the E46K mutation [27]. Homozygous TgM47^+/+^ mice develop spontaneous neurological disease with an onset between 16 and 29 months of age, which is accompanied by accumulation of phosphorylated α-synuclein aggregates in the hindbrain and spinal cord [27]. Alternatively, we observed that hemizygous TgM47^+/-^ mice did not develop neurological signs by 545 days of age (~18 months; S1 Fig) and lack pathological α-synuclein accumulation in the brain, as determined using both our panel of α-syn–YFP cells (S1 Table) and immunostaining of fixed tissue for phosphorylated α-synuclein (S1 Fig). Given this absence of spontaneous disease, we chose to use hemizygous mice for the experiments in this study.

**Fig 1:**
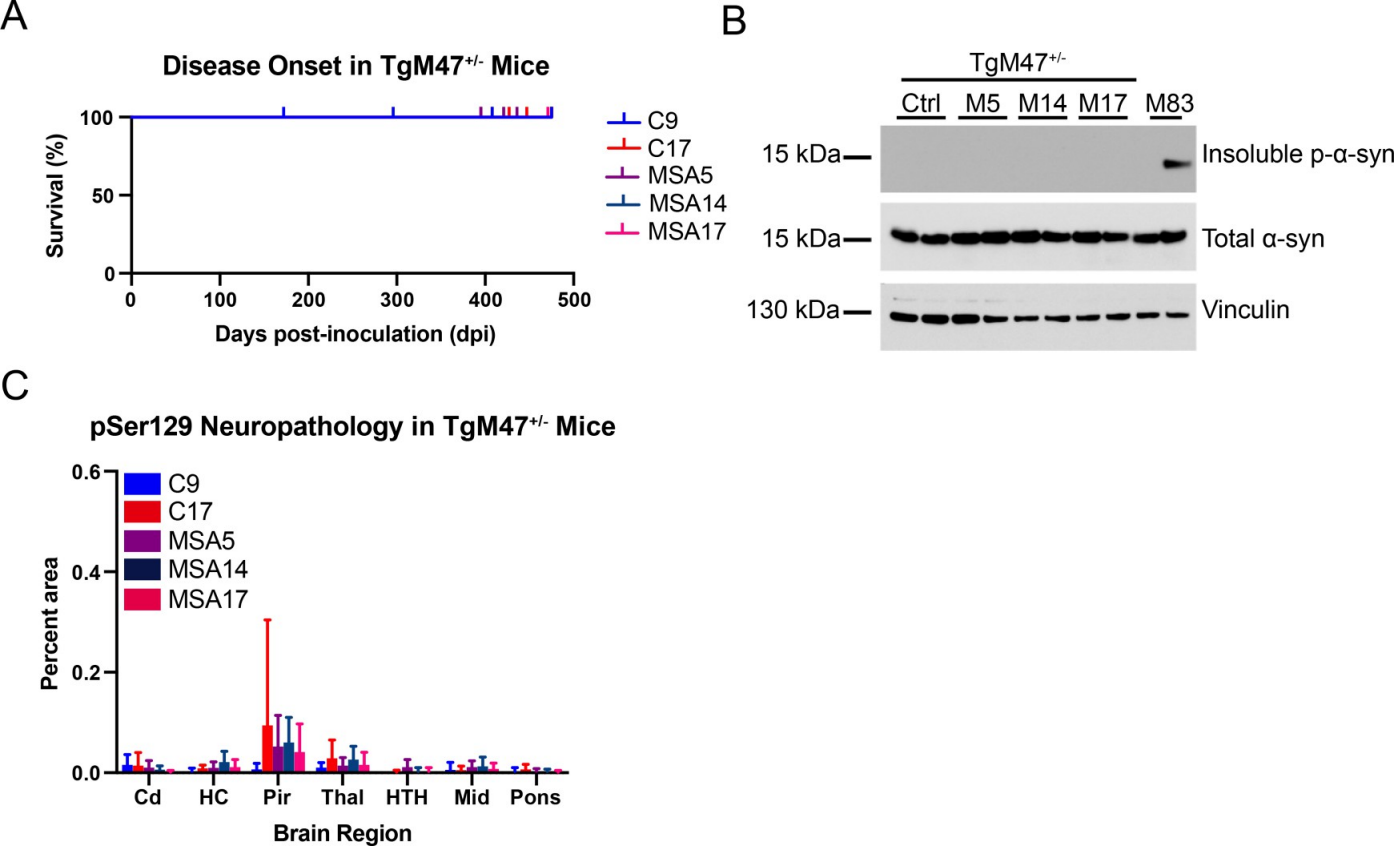
TgM47^+/-^ mice are resistant to MSA prion transmission. Eight-week old TgM47^+/-^ mice were inoculated with either control (C9 and C17) or MSA patient samples (MSA5, MSA14, and MSA17). (A) Kaplan-Meier plot of disease onset in TgM47^+/-^ mice following inoculation. TgM47^+/-^ mice did not develop overt motor signs by 475 days postinoculation (dpi; *P* > 0.05). (B) Two brain homogenates from TgM47^+/-^ mice inoculated with either control or with each of the MSA patient samples were assayed for the presence of insoluble α-synuclein following detergent extraction. An MSA-inoculated TgM83^+/-^ mouse brain, which we have previously shown is positive for phosphorylated α-synuclein, was included as a positive control (EP1536Y primary antibody). None of the homogenates from the inoculated mice contained aggregated α-synuclein, though total α-synuclein levels (MJFR1 primary antibody) were consistent across all homogenates tested. Vinculin shown as a loading control. (C) Fixed half-brains from the same mice were analyzed for phosphorylated α-synuclein neuropathology in the caudate (Cd), hippocampus (HC), piriform cortex and amygdala (Pir), thalamus (Thal), hypothalamus (HTH), midbrain (Mid), and pons. None of the inoculated animals developed detectable pathology (*P* > 0.05).

To determine if MSA prions replicate in Tg mice expressing E46K α-synuclein *in vivo*, we inoculated eight-week-old TgM47^+/-^ mice intracranially (i.c.) with 30 μL 1% brain homogenate prepared from either two control patient samples (C9 and C17) or three MSA patient samples (MSA5, MSA14, and MSA17; S2 Table) and monitored the mice for the onset of neurological signs (Fig 1). Consistent with our *in vitro* findings, all animals remained healthy at 475 dpi, regardless of inocula (Fig 1A; *P* > 0.05). To determine if the MSA-inoculated mice were preclinical at the time of collection, we tested aliquots of frozen half-brains in our panel of α-syn–YFP cell lines and found that none of the samples were able to infect the cell lines tested (Table 1; *P* > 0.05). We were also unable to detect sarkosyl-insoluble α-synuclein in the mouse samples via Western blot, though control- and MSA-inoculated TgM47^+/-^ mice expressed similar levels of total α-synuclein (Fig 1B). Finally, we analyzed fixed half-brains from the same mice for the presence of phosphorylated α-synuclein inclusions and found that neither the control nor the MSA patient samples induced α-synuclein pathology in the TgM47^+/-^ mice (Figs 1C and S2; *P* > 0.05). Together, these findings indicate that MSA prions are unable to replicate *in vivo* using E46K α-synuclein as substrate.

**Table 1:** Infectivity of TgM47^+/-^ passaged MSA prions in cultured cells.

	Mean cell infection ± SD^a^ (Fluorescence/Cell × 10^3^ A.U.)				
Sample	α-syn140-YFP	α-syn140*E46K-YFP	α-syn140*A53T-YFP	α-syn140*E46K,A53T-YFP	α-syn95*A53T-YFP
C9	1.0 ± 0.3	1.1 ± 1.0	1.7 ± 1.0	0.3 ± 0.1	2.0 ± 2.6
C17	0.8 ± 0.2	0.8 ± 0.2	1.0 ± 0.2	0.5 ± 0.1	1.3 ± 0.3
MSA5	0.9 ± 0.3	0.8 ± 0.2	1.4 ± 0.2	0.4 ± 0.1	1.2 ± 0.1
MSA14	0.8 ± 0.1	0.9 ± 0.3	1.4 ± 0.3	0.4 ± 0.1	1.4 ± 0.1
MSA17	1.0 ± 0.1	0.8 ± 0.2	1.3 ± 0.2	0.4 ± 0.1	1.7 ± 0.4

^a^Colors represent heat map of infectivity with blue indicating high infectivity for a given cell line and red indicating low infectivity for a given cell line.

### E46K PFFs replicate slowly in TgM47^+/-^ mice

**Fig 2:**
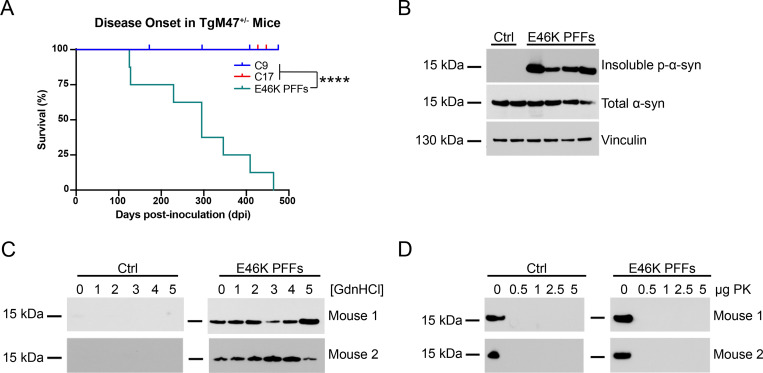
E46K PFFs transmit neurological disease to TgM47^+/-^ mice. Eight-week-old TgM47^+/-^ mice were inoculated with 30 μg of E46K α-synuclein PFFs or 30 μL of 1% brain homogenate from control patient samples. (A) Kaplan-Meier plot shows E46K PFFs induced neurological disease 286 ± 122 days postinoculation (dpi; ****P < 0.0001). (B) Two brain homogenates from TgM47^+/-^ mice inoculated with control patient samples and four homogenates from mice inoculated with E46K PFFs were assayed for the presence of insoluble α-synuclein following detergent extraction. All four homogenates from E46K PFF–inoculated mice contained phosphorylated α-synuclein (EP1536Y primary antibody). All mice tested expressed similar levels of total α-synuclein (MJFR1 primary antibody). Vinculin shown as a loading control. (C) Guanidine hydrochloride (GdnHCl) denaturation was performed using brain homogenates from two control-inoculated (left) and two E46K PFF–inoculated TgM47^+/-^ mice (right) using 0, 1, 2, 3, 4, and 5 *M* GdnHCl. After detergent extraction, α-synuclein in the resulting protein pellet was detected via Western blot with the total α-synuclein antibody MJFR1. (D) Proteinase K (PK) digestion was performed using brain homogenates from two control-inoculated (left) and two E46K PFF–inoculated TgM47^+/-^ mice (right) using 0, 0.5, 1, 2.5, and 5 μg PK. After detergent extraction, total α-synuclein in the resulting protein pellets was detected via Western blot with the antibody MJFR1.

**Fig 3:**
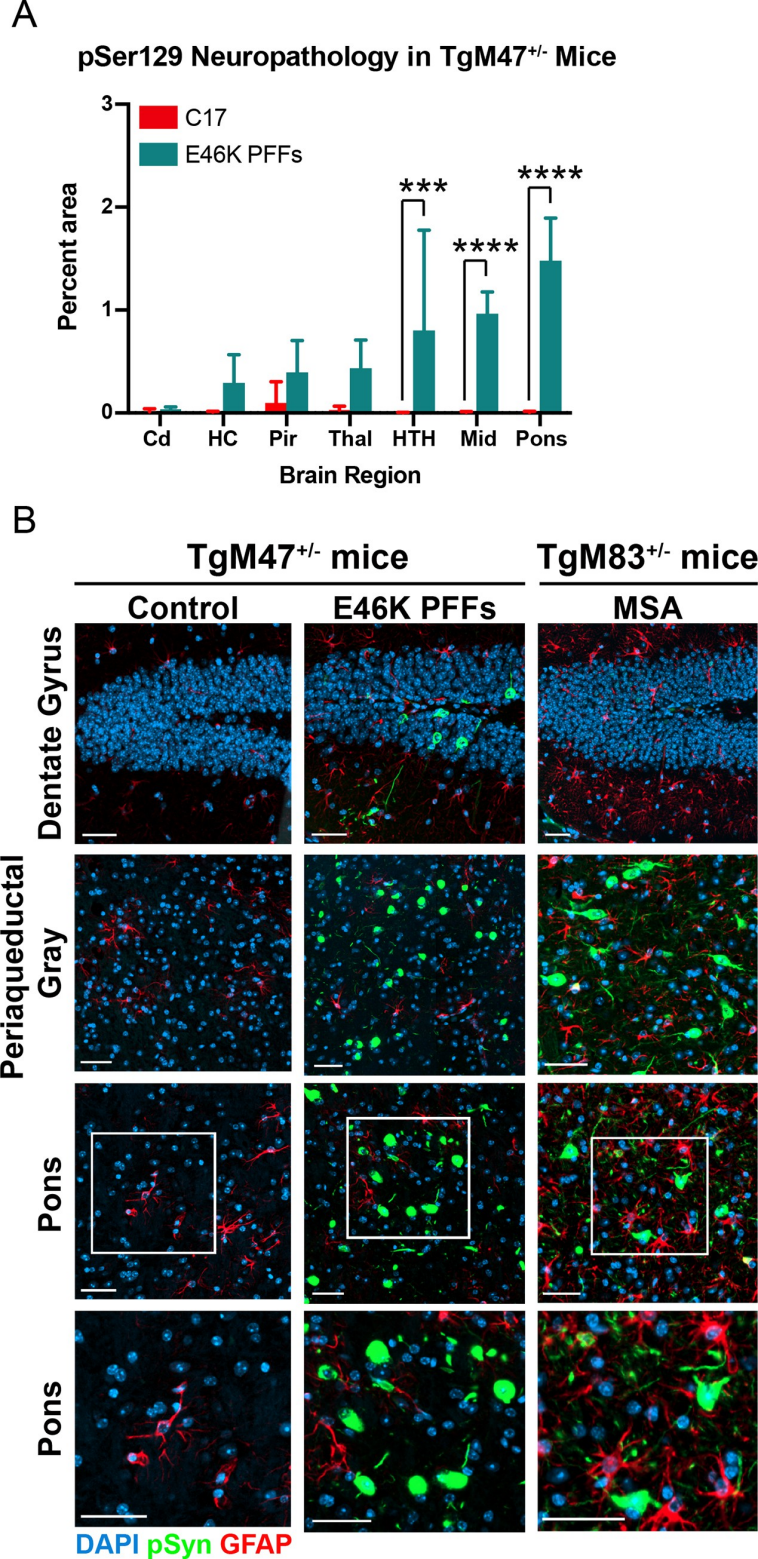
E46K PFFs induce phosphorylated α-synuclein neuropathology in TgM47^+/-^ mice. Fixed tissues from TgM47^+/-^ mice inoculated with the control patient sample C17 or E46K PFFs were analyzed for phosphorylated α-synuclein inclusions (EP1536Y primary antibody). (A) Quantification of neuropathology showed the caudate (Cd) was unaffected by E46K PFF transmission. The hippocampus (HC), piriform cortex and amygdala (Pir), and thalamus (Thal) developed inclusions that were not statistically different from control (*P* > 0.05). However, E46K PFFs induced robust inclusion formation in the hypothalamus (HTH; ***P < 0.001), midbrain (Mid; ****P < 0.0001), and pons (****P < 0.0001). (B) Representative images from TgM47^+/-^ mice inoculated with control patient samples (left column) or E46K PFFs (middle column) as well as an MSA-inoculated TgM83^+/-^ mouse sample shown for comparison (left column). From top to bottom, images of the dentate gyrus, periaqueductal gray, and pons are shown. Region denoted with a white box in the pons is shown at higher magnification in the bottom row. Phosphorylated α-synuclein in green, glial fibrillary acidic protein (GFAP) in red, and DAPI in blue. Scale bar: 50 μm.

To confirm that TgM47^+/-^ mice can develop synucleinopathy following i.c. injection with α-synuclein prions, we inoculated eight-week-old mice with recombinant E46K or WT α-synuclein PFFs. α-Synuclein monomers containing an N-terminal His tag (Sigma Aldrich) were fibrillized in DPBS at 37°C with shaking for 1 week. To first test homotypic fibril transmission, we inoculated TgM47^+/-^ mice with either 30 μL of 1% (wt/vol) brain homogenate from two control patient samples or 30 μg of E46K PFFs (Figs 2 and 3; Table 2). The mice were then evaluated for the onset of neurological signs twice each week. Unlike the control-inoculated animals, TgM47^+/-^ mice inoculated with the E46K PFFs developed neurological signs 286 ± 122 dpi (Fig 2A; *P* < 0.0001). Notably, we observed substantial variability in disease onset in the mice; the first animal developed clinical signs at 125 dpi while the last mouse did not show signs until 464 dpi. Compared to transmission studies in the TgM83^+/-^ mice, where disease onset occurs within 120 ± 26 dpi on average [19,21,40], the observed incubation period in the TgM47^+/-^ mice is both delayed and extended. One possible explanation for these findings is that E46K PFFs propagate with slower kinetics than A53T α-synuclein, as has been shown previously [41]. Supporting this hypothesis, we found that the E46K PFF–inoculated mouse samples were only able to propagate in the E46K,A53T cells when we tested them in the α-syn–YFP cell assay (Table 2; *P* < 0.0001). Therefore, it is likely that, in the presence of both the E46K and A53T mutations, the α-syn–YFP cells can support homotypic templating while also accelerating misfolding kinetics, enabling detection of a slower propagating E46K prion strain.

**Table 2:** Infectivity of TgM47^+/-^ passaged E46K PFFs in cultured cells.

	Mean cell infection ± SD^a^ (Fluorescence/Cell × 103 A.U.)				
Sample	α-syn140-YFP	α-syn140*E46K-YFP	α-syn140*A53T-YFP	α-syn140*E46K,A53T-YFP	α-syn95*A53T-YFP
C9	1.0 ± 0.3	1.1 ± 1.0	1.7 ± 1.0	0.3 ± 0.1	2.0 ± 2.6
C17	0.8 ± 0.2	0.8 ± 0.2	1.0 ± 0.2	0.5 ± 0.1	1.3 ± 0.3
E46K PFFs	0.8 ± 0.1	0.7 ± 0.1	1.3 ± 0.4	4.8 ± 3.7***	1.8 ± 0.4

^a^Colors represent heat map of infectivity with blue indicating high infectivity for a given cell line and red indicating low infectivity for a given cell line.

*** = P < 0.001 compared to control-patient samples C9 and C17.

Biochemical analysis of sarkosyl-insoluble α-synuclein aggregates from mice inoculated with E46K PFFs showed that the pathogenic protein in the clinical animals was phosphorylated at residue S129 (Fig 2B). We also observed consistent levels of total α-synuclein expression across inoculated animals, eliminating the possibility that differences in substrate expression contributed to the varied incubation times seen in the mice. Intriguingly, GdnHCl denaturation did not degrade α-synuclein aggregates, detected using the total α-synuclein antibody MJFR1, in the E46K PFF–inoculated brain homogenates (Fig 2C), but any amount of PK digestion did (Fig 2D). These findings indicate the aggregates are resistant to chaotropic agents while remaining susceptible to proteases; however, it is possible that we would obtain different results using a milder protease, such as thermolysin, or an antibody for a different region of the protein.

Lastly, we used fixed half-brains to assess the ability of E46K PFFs to induce phosphorylated α-synuclein pathology in the TgM47^+/-^ mice (Fig 3). While we observed α-synuclein inclusions in the hippocampus, piriform cortex and amygdala, and thalamus that were not statistically different from control-inoculated animals (*P* > 0.05), the neuropathology present in the hypothalamus, midbrain, and pons was significantly increased compared to the control-injected animals (Fig 3A; *P* < 0.001). Notably, the perinuclear inclusions observed in the E46K PFF–inoculated TgM47^+/-^ mice differ from the pathology observed in MSA-inoculated TgM83^+/-^ mice, which is characterized by the presence of phosphorylated α-synuclein throughout the neuronal cell body and neurites in the hindbrain (Fig 3B). These data demonstrate that E46K PFFs propagate in TgM47^+/-^ mice with delayed kinetics, inducing neurological disease and the formation of mature, phosphorylated inclusions in the brain.

### WT PFFs propagate inefficiently in TgM47^+/-^ mice

**Fig 4:**
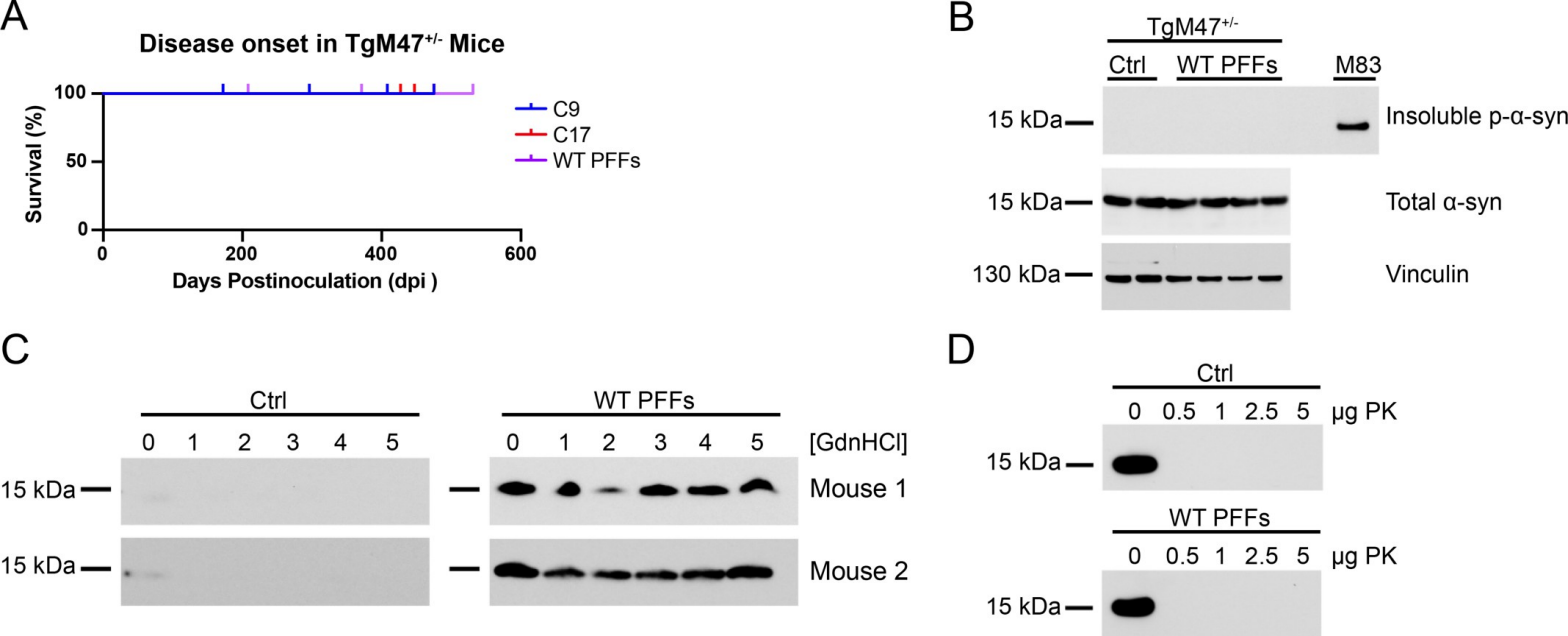
TgM47^+/-^ mice propagate WT α-synuclein PFFs. Eight-week-old TgM47^+/-^ mice were inoculated with 30 μg of WT human α-synuclein PFFs or 30 μL of 1% brain homogenate from control patient samples. (A) Kaplan-Meier plot shows mice inoculated with WT PFFs did not develop neurological disease 531 days postinoculation (dpi; *P* > 0.05). (B) Two brain homogenates from TgM47^+/-^ mice inoculated with control patient samples and four homogenates from mice inoculated with WT PFFs were assayed for the presence of insoluble α-synuclein following detergent extraction. An MSA-inoculated TgM83^+/-^ mouse brain, which we have previously shown is positive for phosphorylated α-synuclein, was included as a positive control (EP1536Y primary antibody). None of the samples from inoculated TgM47^+/-^ mice contained aggregated α-synuclein, though total α-synuclein levels (MJFR1 primary antibody) were consistent across all mice tested. Vinculin shown as a loading control. (C) Guanidine hydrochloride (GdnHCl) denaturation was performed using brain homogenates from two control-inoculated (left) and two WT PFF–inoculated TgM47^+/-^ mice (right) using 0, 1, 2, 3, 4, and 5 *M* GdnHCl. After detergent extraction, α-synuclein in the resulting protein pellet was detected via Western blot with the total α-synuclein antibody MJFR1. (D) Proteinase K (PK) digestion was performed using brain homogenates from one control-inoculated (top) and one WT PFF–inoculated TgM47^+/-^ mouse (bottom) using 0, 0.5, 1, 2.5, and 5 μg PK. After detergent extraction, total α-synuclein in the resulting protein pellets was detected via Western blot with the antibody MJFR1.

Intracerebral injection of recombinant WT fibrils (containing α-synuclein residues 21–140) into the hippocampus of TgM47 mice was previously shown to induce minimal pathology 4 mpi [28]. More recently, studies by Long *et al*. reported that WT fibrils could not nucleate fibril elongation using E46K α-synuclein as substrate [37]. To determine if the E46K mutation exerts a selective pressure on WT PFF propagation *in vivo*, we inoculated eight-week-old TgM47^+/-^ mice with 30 μg of recombinant WT PFFs (Fig 4, Table 3, and S3 Fig). By 531 dpi, none of the inoculated animals developed neurological signs (Fig 4A; *P* > 0.05); however, brain homogenates from WT PFF–inoculated mice contained α-synuclein prions that propagated in the WT and A53T cell lines (Table 3; *P* < 0.0001). In contrast, these samples could not replicate in the E46K, E46K,A53T, or truncated α-syn*A53T (1–95) cell lines (*P* > 0.05).

**Table 3:** Infectivity of TgM47^+/-^ passaged WT PFFs in cultured cells.

	Mean cell infection ± SD^a^ (Fluorescence/Cell × 10^3^ A.U.)				
Sample	α-syn140-YFP	α-syn140*E46K-YFP	α-syn140*A53T-YFP	α-syn140*E46K,A53T-YFP	α-syn95*A53T-YFP
C9	1.0 ± 0.3	1.1 ± 1.0	1.7 ± 1.0	0.3 ± 0.1	2.0 ± 2.6
C17	0.8 ± 0.2	0.8 ± 0.2	1.0 ± 0.2	0.5 ± 0.1	1.3 ± 0.3
WT PFFs	9.7 ± 5.1****	0.1 ± 0.0	21 ± 6.3****	1.5 ± 0.8	4.4 ± 2.8

^a^Colors represent heat map of infectivity with blue indicating high infectivity for a given cell line and red indicating low infectivity for a given cell line.

**** = P < 0.0001 compared to control-patient samples C9 and C17.

Previously, we posited that recombinant PFFs likely exist as a mixture of conformations, some of which propagate better than others under varying selective pressures [22,42]. One result of this variability is that only a fraction of the total conformations present can replicate using E46K α-synuclein as substrate. Consequently, when a mixture of WT PFFs is passaged in TgM47^+/-^ mice, as we tested here, the animal genotype exerts a selective pressure on the inoculum, selecting for replication-competent α-synuclein species as the strain replicates in a new host. This essentially creates a low titer inoculation where the α-synuclein prions replicate but the mice remain in a preclinical stage at the time of collection. Supporting this hypothesis, we observed a change in strain biology following passaging of WT PFFs in the TgM47^+/-^ mice. Prior to inoculation, the WT PFFs propagated in all of the α-syn–YFP cell lines tested [22], but after transmission, replication was only detected in two cell lines (Table 3). Subsequent passaging studies using homogenates from these mice are needed to determine if strain adaptation has occurred.

We have shown that our cell-based models for protein misfolding are more sensitive to the presence of pathogenic protein in a sample than other commonly used detection methods [43,44]. To assess the stage of preclinical WT PFF replication in the TgM47^+/-^ mice, we used biochemistry and immunohistochemistry to characterize the pathogenic α-synuclein present in the brains of the mice. We first used Western blots to probe for the presence of sarkosyl-insoluble phosphorylated α-synuclein isolated from fresh-frozen brain homogenates (Fig 4B). Neither control-inoculated nor WT PFF–inoculated mice contained sarkosyl-insoluble α-synuclein. These results are consistent with the idea that some posttranslational modifications, including phosphorylation, occur after self-templating rather than before [45]. We then used GdnHCl denaturation to detect the presence of GdnHCl-resistant α-synuclein aggregates using an antibody for total α-synuclein (MJFR1; Fig 4C). While we could not detect any GdnHCl-resistant α-synuclein in the brains of control-inoculated mice, the brains of WT PFF–inoculated mice contained GdnHCl-resistant protein aggregates. Notably, similar to data collected from mice inoculated with E46K PFFs, the amount of total α-synuclein remaining in the samples following denaturation with 5 *M* GdnHCl was unchanged from the amount of total α-synuclein following incubation without GdnHCl. These findings indicate that even though we could not detect α-synuclein phosphorylated at residue S129, the insoluble aggregates that are present are highly resistant to chaotropic agents. In contrast, we were unable to detect any α-synuclein resistant to PK digestion in control-inoculated or WT PFF–inoculated animals (Fig 4D). Though, as previously mentioned, these results may be impacted by protease and antibody epitope. Finally, we also analyzed immunostained fixed-tissue sections from the same mice for the presence of α-synuclein neuropathology (S3 Fig). Consistent with our biochemistry experiments, the WT PFFs failed to induce the formation of phosphorylated α-synuclein inclusions. These findings suggest that the WT PFF inoculum contained a low titer of replication-competent conformations of α-synuclein prions.

An important caveat to consider regarding these findings is the possible persistence of inoculum in the mouse brain. However, the altered biological activity of the brain homogenate in the α-syn–YFP cell lines is consistent with strain selection rather than persistent inoculum. Moreover, in the unlikely event that all 30 μg of WT PFFs remained in the brain by 531 dpi, the maximum concentration of WT PFFs incubated with the cells would be ~0.0008 μg/μL. We previously tested titration curves using WT PFFs on our cell lines and found the detectable limit is ~0.25 μg/μL [22], indicating that infection in the α-syn–YFP cells cannot be due to the presence of persistent inoculum in the absence of replication.

### E46K PFF replication kinetics are slowed when using WT α-synuclein as substrate

**Fig 5:**
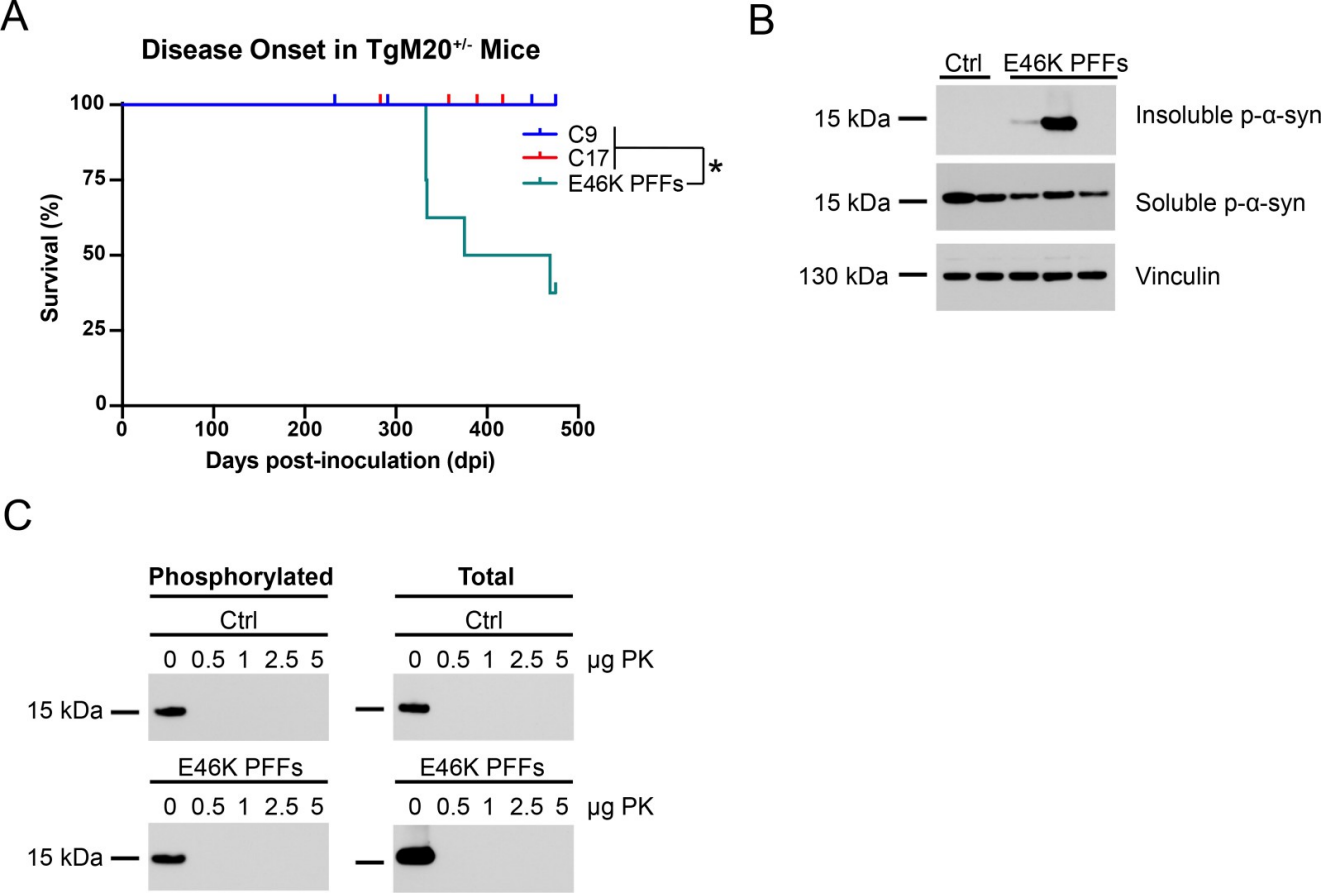
TgM20^+/-^ mice propagate E46K α-synuclein PFFs. Eight-week-old TgM20^+/-^ mice were inoculated with 30 μg of E46K human α-synuclein PFFs or 30 μL of 1% brain homogenate from control patient samples. (A) Kaplan-Meier plot shows five of eight mice inoculated with E46K PFFs developed neurological disease by 475 days postinoculation (dpi; *P* > 0.05). Incubation times for control-inoculated mice previously reported in Holec, *et al*., 2022 [46]. (B) Two brain homogenates from TgM20^+/-^ mice inoculated with control patient samples and three homogenates from mice inoculated with E46K PFFs were assayed for the presence of insoluble α-synuclein following detergent extraction (EP1536Y primary antibody). Some of the samples from E46K PFF–inoculated TgM20^+/-^ mice contained aggregated α-synuclein (EP1536Y primary antibody), though soluble α-synuclein was present in all mice tested. Vinculin shown as a loading control. (C) Proteinase K (PK) digestion was performed using brain homogenates from control-inoculated and E46K PFF–inoculated TgM20^+/-^ mice using 0, 0.5, 1, 2.5, and 5 μg PK. After detergent extraction, phosphorylated and total α-synuclein in the resulting protein pellets were detected via Western blot using both EP1536Y (left) and MJFR1 (right) primary antibodies, respectively.

Considering our finding that WT PFFs inefficiently replicate when forced to use E46K α-synuclein as substrate, we next sought to determine how propagation of E46K PFFs is impacted during heterotypic replication. In these experiments, we used the TgM20^+/-^ mouse model that uses the *Prnp* promoter to express WT human α-synuclein [17], making this the best model available to test this effect. We recently showed that both WT PFFs and MSA patient samples propagate with high fidelity in TgM20^+/-^ mice, inducing neurological disease over an extended incubation period compared to transmission studies in the TgM83^+/-^ mouse model [46]. Here, eight-week-old TgM20^+/-^ mice inoculated with 30 μL of 1% brain homogenate from control patient samples or 30 μg E46K PFFs were assessed biweekly for the onset of neurological signs. While none of the control-inoculated mice developed disease, five of the eight mice inoculated with E46K PFFs developed disease before the experiment concluded at 475 dpi (Fig 5A; *P* < 0.05). Unexpectedly, α-synuclein prions isolated from mice inoculated with E46K PFFs propagated in the WT, A53T, E46K,A53T, and truncated α-syn*A53T (1–95) cell lines (*P* < 0.0001) but not in the E46K cells (Table 4; *P* > 0.05). Moreover, two of the healthy mice euthanized at 475 dpi were positive for α-synuclein prions, indicating preclinical propagation in the animals. These data suggest that the kinetics of E46K PFF propagation are slowed when forced to use WT α-synuclein as substrate.

**Table 4:** Infectivity of TgM20^+/-^ passaged E46K PFFs in cultured cells.

	Mean cell infection ± SD^a^ (Fluorescence/Cell × 10^3^ A.U.)				
Sample	α-syn140-YFP	α-syn140*E46K-YFP	α-syn140*A53T-YFP	α-syn140*E46K,A53T-YFP	α-syn95*A53T-YFP
C9	1.0 ± 0.3	1.0 ± 0.2	2.4 ± 1.1	0.8 ± 0.9	2.1 ± 1.1
C17	1.0 ± 0.5	0.9 ± 0.2	2.2 ± 1.3	0.4 ± 0.2	1.7 ± 1.2
E46K PFFs	13 ± 6.6****	0.9 ± 0.9	18 ± 7.3****	9.6 ± 4.5****	8.4 ± 6.6****

^a^Colors represent heat map of infectivity with blue indicating high infectivity for a given cell line and red indicating low infectivity for a given cell line. Cell infection data from mice inoculated with samples C9 and C17 reported in Holec, et al., 2022 [46].

**** = P < 0.0001 compared to control-patient samples C9 and C17.

We next used biochemical assays to characterize the pathogenic α-synuclein in the resulting brain homogenates (Fig 5B and 5C). Sarkosyl-extracted protein pellets from control-inoculated mice did not contain phosphorylated α-synuclein, but some of the pellets from E46K PFF–inoculated mice did (Fig 5B). We then used PK to determine if the E46K prions were resistant to digestion after passaging in TgM20^+/-^ mice. We first probed blots using the EP1536Y primary antibody to detect α-synuclein phosphorylated at residue S129 and found that bands were only present in undigested samples (Fig 5C, left). Moreover, blots probed using the MJFR1 primary antibody for total α-synuclein also failed to detect PK-resistant α-synuclein (Fig 5C, right), indicating that the E46K strain loses PK resistance after passaging in Tg mice. It is possible, however, that α-synuclein fibrils in these mice are resistant to milder proteases or are detectable using antibodies with epitopes directed to other regions of the protein.

**Fig 6:**
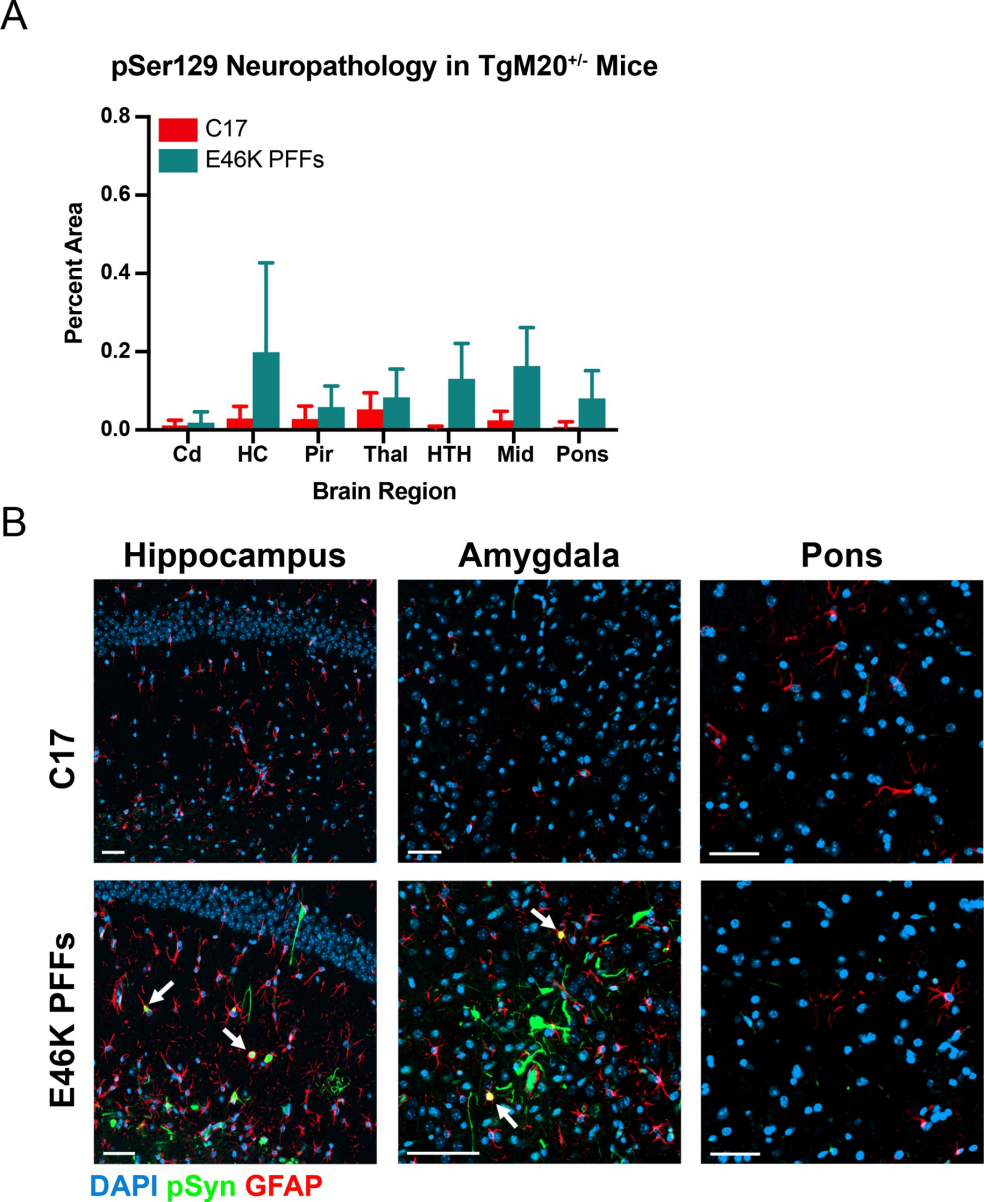
TgM20^+/-^ mice inoculated with E46K PFFs develop mild phosphorylated α-synuclein neuropathology. Fixed tissues from TgM20^+/-^ mice inoculated with control patient sample C17 or E46K PFFs were immunostained for phosphorylated α-synuclein (EP1536Y primary antibody) and glial fibrillary acidic protein (GFAP: astrocytes). (A) Quantification of stained brain slices showed no significant phosphorylated α-synuclein inclusions were present in the caudate (Cd), hippocampus (HC), piriform cortex and amygdala (Pir), thalamus (Thal), hypothalamus (HTH), midbrain (Mid), or pons (*P* > 0.05). Neuropathology data for control-inoculated mice was previously reported in Holec, *et al*., 2022 [46]. (B) Representative images of the HC, amygdala, and pons from mice inoculated with either control patient sample C17 (top) or E46K PFFs (bottom). Notably, some mice did develop some inclusions in the HC and amygdala. While these inclusions appeared to be mostly neuronal, overlap with GFAP staining suggests there may also be colocalization with astrocytes. Phosphorylated α-synuclein in green, GFAP in red, and DAPI in blue. Scale bar: 50 μm.

Finally, we analyzed immunostained fixed sections for the presence of α-synuclein neuropathology (Fig 6). The E46K PFFs failed to induce any significant neuropathological inclusions in the TgM20^+/-^ mice (Fig 6A). However, we did observe some phosphorylated α-synuclein immunostaining in the hippocampus and amygdala in the four brains available for analysis from the five clinical mice (Fig 6B). These findings are consistent with the variable presence of insoluble phosphorylated α-synuclein detected via Western blot (Fig 5B). Notably, while the majority of the impacted cells appeared to be neuronal, we did also observe overlap with GFAP staining, suggesting there may be colocalization with astrocytes (arrows in Fig 6B). Together, these findings indicate that when E46K PFFs are forced to use WT α-synuclein as substrate, replication kinetics are delayed.

## Discussion

The E46K mutation in α-synuclein was first identified in a Spanish family with autosomal dominant PD and DLB [7]. These patients exhibited early onset of severe parkinsonism and dementia in addition to visual hallucinations. The point mutation is located in the fourth of seven imperfect KTKEGV repeats, which are thought to support α-synuclein’s interactions with lipids [47]. Until the recent discoveries of the T72M and E83Q mutations [13,14], E46K was the only α-synuclein mutation known to disrupt one of the KTKEGV repeats, suggesting it may exert differing effects on α-synuclein biology and pathogenesis than other PD-causing mutations. Studies investigating the effect of the E46K mutation on α-synuclein fibrillization kinetics found that the amino acid substitution increases the protein’s propensity to fibrillize compared to WT α-synuclein [41]. Interestingly, this increase in kinetics is not as significant as the effect of the PD-causing A53T mutation. Consistent with these data, disease onset in TgM47^+/+^ mice is delayed compared to the TgM83^+/+^ mouse model [27]. However, both of these models develop a spontaneous synucleinopathy, whereas the TgM20^+/+^ mice—which express WT human α-synuclein—do not.

We previously compared the ability of MSA α-synuclein prions to propagate in cultured cells using WT, E46K, and A53T α-synuclein as substrate [22]. While the MSA patient samples easily replicated in HEK293T cells expressing WT and A53T α-syn–YFP fusion proteins, the presence of the E46K mutation inhibited *in vitro* propagation. Cryo-EM structures of misfolded α-synuclein fibrils isolated from MSA patient samples, as well as many recombinant WT fibrils (which misfold into conformations that differ from those seen in MSA patients), contain a Greek key motif that is stabilized by a salt bridge between residues E46 and K80 [30–34]. We, and others, have hypothesized that the E46K mutation results in a repulsion between the two lysines at residues 46 and 80, which interferes with MSA prion propagation *in vitro* [22,34,42]. Consistent with this hypothesis, cryo-EM structures of recombinant fibrils made using E46K α-synuclein lack the E46/K80 salt bridge; instead, the protein adopts an alternate conformation that is stabilized by a salt bridge between residues K45 and E57 [35]. Together, these structural and biological data suggest that MSA prions cannot use E46K α-synuclein as a substrate for templating. In comparison, the recently resolved structure of α-synuclein fibrils isolated from LBD patients show the conformation is stabilized by a salt bridge between residues E35 and K80, leaving E46 to project outward [39]. While it is not immediately obvious from this structure how the E46K mutation impacts α-synuclein fibrillization in LBD patients, it is clear that the mutation does not disrupt or interfere with protein misfolding.

To test the hypothesis that the E46K mutation inhibits *in vivo* propagation of MSA prions, we inoculated TgM47^+/-^ mice—which do not develop spontaneous disease (S1 Fig and S1 Table)—with brain homogenates prepared from deceased MSA patient samples. We previously reported that MSA inoculations induce neurological disease in ~120 dpi in the TgM83^+/-^ mouse model [18,19,21,40] and in ~281 dpi in the TgM20^+/-^ model [46]. However, the same MSA samples were unable to transmit disease to the TgM47^+/-^ animals by 475 dpi (Fig 1, Table 5). Moreover, we were unable to detect the presence of preclinical MSA prion propagation in the brains of the inoculated mice using multiple assays on either frozen or fixed brain tissue from the animals (Fig 1, Table 1, and S2 Fig). These findings demonstrate that the *in vitro* α-syn–YFP cell assays are predictive of *in vivo* strain biology. Given that the cell assay can be run in 4 days while the animal bioassay can require up to 500 days, more widespread use of these cell lines will accelerate research investigating strain biology. Additionally, these data underscore the conformational, or strain, differences between the α-synuclein prions responsible for disease in MSA patients and those responsible for disease in patients harboring the E46K mutation. These critical differences argue for the need to incorporate strain specificity into synucleinopathy research to better facilitate successful translation of discoveries into the clinic.

**Table 5:** Summary of transmission studies in mice expressing E46K or WT α-synuclein.

Inoculum	Mouse Model		Cell Infection	pSer129 Neuropathology
	Line	Clinical Signs		
Control	TgM47^+/-^ (E46K)	No	No	No
Control	TgM20^+/-^ (WT)	No	No	No
MSA	TgM47^+/-^ (E46K)	No	No	No
MSA^a^	TgM20^+/-^ (WT)	Yes	Yes	Yes
E46K PFFs	TgM47^+/-^ (E46K)	Yes	E46K,A53T only	Yes
E46K PFFs	TgM20^+/-^ (WT)	5 of 8 mice	Yes	Mild
WT PFFs	TgM47^+/-^ (E46K)	No	Yes	No
WT PFFs^a^	TgM20^+/-^ (WT)	Yes	Yes	Yes

^*a*^ *Data published in Holec et al, 2022 [46], and included here for comparison.*

Unlike mice inoculated with MSA samples, TgM47^+/-^ mice inoculated with E46K PFFs developed neurological signs, sarkosyl-insoluble hyperphosphorylated aggregates, and α-synuclein neuropathology (Figs 2 and 3, Tables 2 and 5). However, the extensive incubation period for these transmissions, compared to studies using the TgM83^+/-^ mice, suggests the E46K mutation exhibits slower misfolding kinetics than the A53T mutation. These findings are consistent with data showing that E46K α-synuclein does not fibrillize as readily as A53T α-synuclein [41] and that disease onset in the TgM83^+/+^ mice occurs earlier than in the TgM47^+/+^ animals [27]. The effect of the relatively slower misfolding kinetics is particularly noticeable in the cell assay data from mice inoculated with E46K PFFs (Table 2). The finding that the α-synuclein prions could only propagate in cells containing both the E46K and A53T mutations is reminiscent of the nucleus size (n_s_) studies on yeast prions by Tricia Serio’s group [48]. This work showed that for yeast strains to propagate, the fibril size must exceed a minimum n_s_ prior to budding, otherwise the fibril becomes unstable and the amyloid disassembles. In our cell model, HEK293T cells double approximately once each day. While strains with faster self-templating kinetics will be able to exceed n_s_ prior to cell division, strains with slower kinetic properties will likely fall below n_s_ and the infection will not be maintained. This may be partially rescued by using an α-syn–YFP construct with mutations that accelerate misfolding kinetics, as was observed here using the E46K,A53T cells.

Notably, the neuropathological inclusions present in E46K PFF–inoculated TgM47^+/-^ mice differed substantially from the pathology we previously reported in MSA-inoculated TgM83^+/-^ animals (Fig 3B). The MSA patient samples induce inclusions throughout neurites in addition to inclusions that fill the entire neuronal cell body. However, most of the pathology seen in TgM47^+/-^ mice inoculated with E46K PFFs can be characterized as round perinuclear inclusions similar in shape to Lewy bodies in PD patients. This is consistent with the conclusion that α-synuclein misfolds into distinct conformations, or strains, in MSA and in PD patients carrying the E46K mutation.

While we have shown incompatibility between MSA prions and E46K α-synuclein *in vivo*, Long *et al*. recently reported that fibrils made from recombinant WT α-synuclein are unable to nucleate fibril extension using E46K α-synuclein as substrate [37]. Cryo-EM structures suggest this is also due to the presence of an E46/K80 salt bridge in the WT fibrils, which prevents the E46K protein from adopting the WT fibril conformation. Similarly, Sacino *et al*. observed that hippocampal inoculations using WT fibrils (made using α-synuclein residues 21–140) into TgM47^+/-^ mice only induced mild, localized neuropathology in the animals, whereas the same inoculation in TgM83^+/-^ mice induced extensive, widespread inclusions 4 mpi [28]. Our finding of preclinical α-synuclein prion propagation in TgM47^+/-^ mice inoculated with WT PFFs is consistent with both of these observations (Fig 4, Table 3, Table 5, and S3 Fig). Cryo-EM studies have shown that WT fibrils can adopt a variety of possible conformations, some of which do not contain the E46/K80 salt bridge [30–33,49,50]. Therefore, within a heterogenous population of WT PFFs, there are likely two pools of conformations: one that can use E46K α-synuclein as substrate and one that cannot. Given this mixture, we would expect incubation time and α-synuclein prion content in TgM47^+/-^ mice to reflect the prevalence of each conformational pool. For example, if the pool predominantly contained WT PFFs lacking the E46/K80 salt bridge, disease onset in TgM47^+/-^ mice would be earlier and end-stage animals would contain more α-synuclein prions. However, we observed no neurological signs in inoculated mice, and the α-synuclein prions we detected were not hyperphosphorylated, indicating the presence of immature inclusions. Consistent with this interpretation, we were unable to detect α-synuclein neuropathology in the inoculated mice. These results suggest that the WT PFFs we generated for inoculation contained a small pool of conformations lacking the E46/K80 salt bridge. This is a notable distinction from the MSA inoculation studies where all known α-synuclein fibril structures in MSA patients contain the E46/K80 salt bridge [34]. As a result, MSA prions are unable to propagate using E46K α-synuclein as substrate, indicating that WT PFFs and MSA patient samples are distinct α-synuclein strains.

Finally, we inoculated TgM20^+/-^ mice with E46K PFFs and observed a delay in disease onset with an incomplete attack rate (Fig 5, Table 5). Notably, two of the healthy mice euthanized at 475 dpi did contain α-synuclein prions in their brains, which is indicative of preclinical prion propagation. Unlike TgM47^+/-^ mice inoculated with E46K PFFs, neuropathological inclusions in the inoculated TgM20^+/-^ mice were rare, limiting our ability to assess their distribution and appearance (Fig 6). However, using our cell assay, we found that E46K PFFs passaged in TgM20^+/-^ mice were able to propagate in all α-syn–YFP cells except those expressing the E46K mutation alone (Table 4). These results indicate that the E46K strain replicated with slower kinetics when WT α-synuclein was available as substrate. This is consistent with the lack of PK-resistant α-synuclein in the samples (Fig 5C), compared to our previous findings that E46K PFFs are resistant to 5 μg PK [22]. These findings may shed important light on the process of α-synuclein propagation in patients harboring a heterozygous E46K mutation.

In summary, we have shown that the presence of the E46K mutation, either in the host or the agent, exerts a selective pressure on α-synuclein prion replication. Most notably, this mutation completely inhibits MSA prions from replicating *in vivo*, whereas preclinical propagation of WT PFFs in TgM47^+/-^ mice indicates that WT PFFs and MSA patient samples must contain distinct α-synuclein strains. Homotypic seeding (i.e., E46K PFFs inoculated into mice expressing E46K α-synuclein) results in disease transmission. By comparison, heterotypic seeding of the E46K PFFs into mice expressing WT human α-synuclein delays transmission kinetics. While an important caveat to these studies is that differences in inoculum titer may impact incubation period in both transgenic mouse models, these findings contribute to a greater understanding of the strain differences underlying MSA and PD as well as the mechanisms by which selective pressures impact α-synuclein strain propagation.

## Materials and methods

### Ethics statement

Animals were maintained in an AAALAC-accredited facility in compliance with the *Guide for the Care and Use of Laboratory Animals*. All procedures used in this study were approved by the University of California, San Francisco, Institutional Animal Care and Use Committee (IACUC) under approved protocol AN182216. Frozen brain tissue samples from neuropathologically confirmed cases of MSA were provided by the Parkinson’s UK Brain Bank at Imperial College London and the Massachusetts Alzheimer’s Disease Research Center. Control patient tissue was provided by Dr. Deborah Mash (Miami Brain Bank). UCSF Institutional Review Board approval was not required for this study. All human samples were anonymized. Demographic information about samples used are included in S2 Table.

### Mice

All animals were housed under ABSL-2 conditions in an environmentally controlled room (10–15 air changes per hour) at a 22.5°C ± 1.4°C temperature, a 45% ± 15% relative humidity, and a 12-hour light/dark cycle. Animals had free access to a Tekland diet from Envigo (Indianapolis, IN) and tap water. Mice were group housed unless an animal’s health status necessitated individual housing. The B6;C3-Tg(*Prnp*-SNCA*E46K)47Gia (referred to here as TgM47^+/-^) and B6;C3-Tg(*Prnp-SNCA*)20Vle (referred to here as TgM20^+/-^) mice were kindly provided by Dr. Benoit Giasson (University of Florida).

### Inoculations

Fresh-frozen human tissue was used to create a 10% (wt/vol) homogenate using calcium- and magnesium-free 1× Dulbecco’s phosphate buffered saline (DPBS) using the Omni Tissue Homogenizer (Omni International). Homogenates were diluted to 1% using 5% (wt/vol) bovine serum albumin in 1× DPBS. Recombinant WT and E46K α-synuclein was aggregated in 1× DPBS as previously described [22]. Fibrils were diluted in 1× DPBS to a final concentration of 1 mg/mL.

Prior to inoculation, eight-week-old TgM47^+/-^ and TgM20^+/-^ mice were anesthetized with isoflurane. Freehand inoculations were performed using 30 μL of the 1% brain homogenate or 1 mg/mL fibrils in the thalamus. Following inoculation, all mice were assessed twice each week for the onset of neurological signs based on standard diagnostic criteria for prion disease [51]. Uninoculated mice were euthanized at 545 days of age. Fibril-inoculated TgM47^+/-^ mice were euthanized 531 days postinoculation (dpi) or following the onset of progressive neurological signs. Control- and MSA-inoculated TgM47^+/-^ mice were euthanized at 475 dpi. TgM20^+/-^ mice were euthanized following onset of progressive neurological signs or at 475 dpi. Following euthanasia, the brain was removed and bisected down the midline. The left hemisphere was frozen for reporter cell assay and biochemical analysis, and the right hemisphere was fixed in formalin for neuropathological assessment.

### α-Synuclein prion quantification assay

Frozen brain tissue was used to make a 10% (wt/vol) brain homogenate in calcium- and magnesium-free 1× DPBS using the Omni Tissue Homogenizer with disposable soft tissue tips (Omni International). Aggregated protein was isolated from the homogenates using phosphotungstic acid (PTA; Sigma) as described [21,52]. Isolated protein pellets were diluted 1:10 in 1× DPBS before testing in the α-synuclein prion quantification assays. HEK293T cells expressing α-syn140–YFP (WT), α-syn140*E46K–YFP (E46K), α-syn140*A53T–YFP (A53T), α-syn140*E46K,A53T–YFP (E46K,A53T), and α-syn95*A53T–YFP (1–95) were generated and cultured as previously reported, and assay conditions were used as described [22]. Cells incubated with isolated protein pellets were imaged using the IN Cell Analyzer 6000 (GE Healthcare). DAPI and FITC images from five different regions in each well were analyzed using IN Cell Developer software with an algorithm designed to quantify intracellular aggregates, represented as total fluorescence per cell (× 10^3^; arbitrary units: A.U.). One measurement was generated for each well across the five regions imaged, and each sample was tested in six replicate wells.

### Immunohistochemistry and neuropathology

Formalin-fixed mouse half-brains were cut into four sections prior to processing through graded alcohols, clearing with xylene, infiltrating with paraffin, and embedding. Thin sections (8 μm) were cut, collected on slides, deparaffinized, and exposed to heat-mediated antigen retrieval with citrate buffer (0.1 *M*, pH 6) for 20 min. Slides were stained using the Thermo Fisher 480S Autostainer with 30 min blocking in 10% (vol/vol) normal goat serum and incubating with primary and secondary antibodies (2 h each). Primary antibodies used include phosphorylated (S129) α-synuclein (EP1536Y; 1:1,000; Abcam) and glial fibrillary acidic protein (GFAP; 1:500; Abcam). Secondary antibodies conjugated to AlexaFluor 488 or 647 (1:500; Thermo Fisher) were used. Slides were imaged using the Zeiss AxioScan.Z1 and were then analyzed using the ZEN Analysis software package (Zeiss). To quantify α-synuclein neuropathology, a pixel intensity threshold was determined using a positive control slide. Regions of interest were drawn around the caudoputamen, hippocampus, piriform cortex and amygdala, thalamus, hypothalamus, midbrain, and pons. The percentage of pixels positive for staining in each region was determined and averaged across inoculation groups.

### Guanidine hydrochloride denaturation

Guanidine hydrochloride (GdnHCl) denaturation of brain homogenates was performed as previously reported [22]. The resulting protein pellets were resuspended in 40 μL 1× NuPAGE LDS loading buffer containing β-mercaptoethanol (20 μL for 5 *M* samples) and were boiled for 10 min prior to immunoblotting.

### Proteinase K digestion

Proteinase K (PK) digestion of brain homogenates was performed as previously reported [22]. Final protein pellets were resuspended in 50 μL 1× NuPAGE LDS loading buffer containing β-mercaptoethanol and boiled for 10 min prior to immunoblotting.

### Immunoblotting

To visualize soluble α-synuclein expression in TgM47^+/-^ and TgM20^+/-^ mice, brain homogenates were clarified by centrifugation for 5 min at 1,000 × g. The supernatant was collected, and total protein was measured using the bicinchoninic acid (BCA) assay (Pierce). A total of 2.5 μg total protein was prepared in 1× NuPAGE LDS loading buffer and boiled for 10 min. Samples were loaded onto a 10% Bis-Tris gel and SDS-PAGE was performed using the MES buffer system. Protein was transferred to a polyvinylidene fluoride (PVDF) membrane, and the membrane was fixed in 0.4% formalin for 30 min at room temperature. The membrane was then incubated in blocking buffer (5% [wt/vol] nonfat milk in 1× Tris-buffered saline containing 0.05% [vol/vol] Tween 20 [TBST]) for 30 min at room temperature. TgM47^+/-^ blots were incubated with primary antibody for total α-synuclein (MJFR1; 1:12,500; Abcam) and the loading control vinculin (1:10,000; Abcam) in blocking buffer overnight at 4°C in a vacuum-sealed pouch. TgM20^+/-^ blots were incubated with primary antibody for phosphorylated (S129) α-synuclein (EP1536Y; 1:4,000; Abcam) and the loading control vinculin (1:10,000) in blocking buffer overnight at 4°C in a vacuum-sealed pouch. Membranes were washed three times with 1× TBST before incubating with horseradish peroxidase–conjugated goat anti-rabbit secondary antibody (1:10,000; Abcam) diluted in blocking buffer for 1 h at 4°C in a vacuum-sealed pouch. After washing blots three times in 1× TBST, membranes were developed using the enhanced chemiluminescent detection system (Pierce) for exposure to X-ray film.

To detect insoluble phosphorylated α-synuclein, protein aggregates were isolated via PTA precipitation, and resuspended pellets were diluted 1:5 in 1× NuPAGE LDS loading buffer and 1× DPBS. Samples were boiled for 20 min before loading on a 10% Bis-Tris gel. Using the protocol described above, PVDF membranes were probed for phosphorylated (S129) α-synuclein (EP1536Y; 1:4,000). A positive control (MSA-inoculated TgM83^+/-^ mouse sample) was used to show successful protein transfer when warranted. Following guanidine hydrochloride (GdnHCl) denaturation and PK digestion, protein pellets were probed for total α-synuclein using the MJFR1 primary antibody (1:10,000). PK-digested pellets from TgM20^+/-^ mice were probed for phosphorylated (S129) α-synuclein using the EP1536Y primary antibody (1:4,000). For insoluble, GdnHCl, and PK blots from TgM47^+/-^ mice, the membrane was not fixed prior to incubating in blocking buffer.

### Statistical analysis

Data are presented as mean ± standard deviation. Data were analyzed using GraphPad Prism software. Kaplan-Meier curves were analyzed using a log-rank Mantel-Cox test. Analysis of data collected from the α-syn–YFP cell assays and neuropathology data comparing control- and MSA-inoculated samples was performed using a two-way ANOVA with a Tukey multiple comparison post hoc test. Neuropathology data comparing control- and fibril-inoculated mice were analyzed using a two-way ANOVA with a Bonferroni multiple comparison post hoc test. Significance was determined with a *P*-value < 0.05.

## Supporting information

S1 Fig Hemizygous TgM47^+/-^ mice do not develop spontaneous disease. (A) TgM47^+/-^ mice were aged to 545 days (~18 months) and were assessed for neurological signs twice each week. However, as shown in the Kaplan–Meier plot, the mice did not develop the signs reported in homozygous TgM47^+/+^ mice [27]. (B) Formalin-fixed half-brains from TgM47^+/-^ mice collected at 545 days of age were immunostained for phosphorylated α-synuclein. Neuropathology was quantified (percent area) in the caudate (Cd), hippocampus (HC), piriform cortex and amygdala (Pir), thalamus (Thal), hypothalamus (HTH), midbrain (Mid), and pons. None of the aged mice developed spontaneous α-synuclein pathology. (C) Representative image showing the lack of phosphorylated α-synuclein pathology in the pons of an aged mouse. DAPI in blue, phosphorylated α-synuclein (EP1536Y primary antibody) in green, and glial fibrillary acidic protein (GFAP: astrocytes) in red. Scale bar: 50 μm.
(TIF)

S2 Fig MSA prions do not induce neuropathological inclusions in TgM47^+/-^ mice. Fixed half-brains from TgM47^+/-^ mice inoculated with either control (C9 or C17) or MSA (MSA5, MSA14, or MSA17) patient samples were collected 475 days postinoculation. Sections were immunostained using the EP1536Y (phosphorylated α-synuclein in green) and GFAP (red) primary antibodies. DAPI shown in blue. Neither the (A) control- nor the (B) MSA-inoculated mice developed α-synuclein inclusions. Scale bar: 50 μm.
(TIF)

S3 Fig TgM47^+/-^ mice inoculated with wild-type (WT) preformed fibrils (PFFs) do not develop phosphorylated α-synuclein neuropathology. Fixed tissues from TgM47^+/-^ mice inoculated with a control patient sample (C17) collected 475 days postinoculation (dpi) or WT PFFs collected 531 dpi were immunostained for phosphorylated α-synuclein (EP1536Y primary antibody) and GFAP. (A) Quantification of stained brain slices showed no phosphorylated α-synuclein inclusions were present in the caudate (Cd), hippocampus (HC), piriform cortex and amygdala (Pir), thalamus (Thal), hypothalamus (HTH), midbrain (Mid), or pons. (B) Representative images of the pons from mice inoculated with either control patient sample C17 (left) or WT PFFs (right). Phosphorylated α-synuclein in green, GFAP in red, and DAPI in blue. Scale bar: 50 μm.
(TIF)

S1 Table Infectivity of aged TgM47^+/-^ samples in cultured cells. (DOCX)

S2 Table Patient sample information. (DOCX)

S1 Text Supplementary materials and methods. (DOCX)
