# Supplementary figures and images for: The E46K mutation modulates α-synuclein prion replication in transgenic mice
Source: PLoS Pathog. 2022 Dec 1;18(12):e1010956. doi: 10.1371/journal.ppat.1010956 (PMC9714912; doi:10.1371/journal.ppat.1010956)

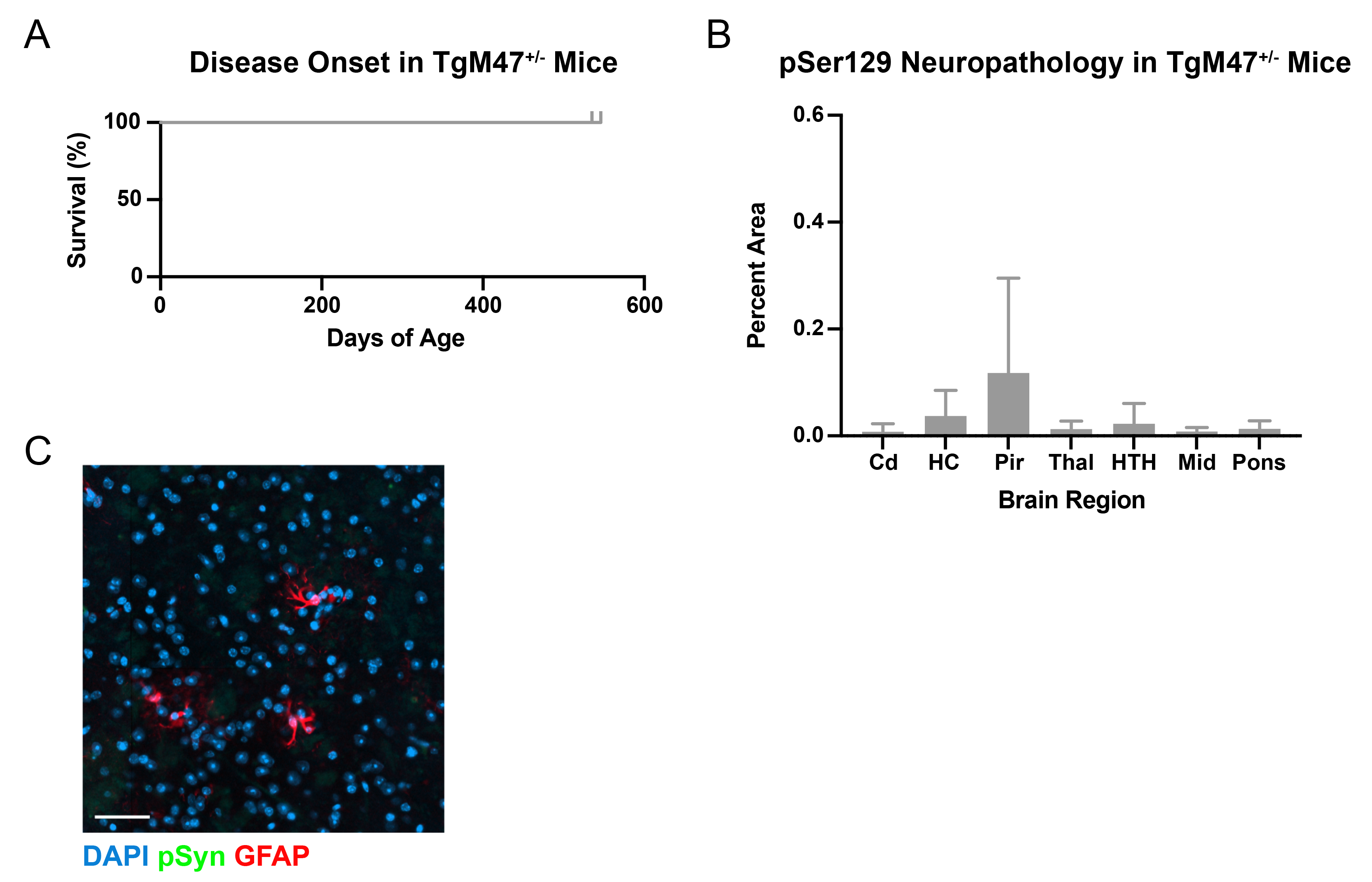

Supplement: S1 Fig — (A) TgM47+/- mice were aged to 545 days (~18 months) and were assessed for neurological signs twice each week. However, as shown in the Kaplan–Meier plot, the mice did not develop the signs reported in homozygous TgM47+/+ mice [27]. (B) Formalin-fixed half-brains from TgM47+/- mice collected at 545 days of age were immunostained for phosphorylated α-synuclein. Neuropathology was quantified (percent area) in the caudate (Cd), hippocampus (HC), piriform cortex and amygdala (Pir), thalamus (Thal), hypothalamus (HTH), midbrain (Mid), and pons. None of the aged mice developed spontaneous α-synuclein pathology. (C) Representative image showing the lack of phosphorylated α-synuclein pathology in the pons of an aged mouse. DAPI in blue, phosphorylated α-synuclein (EP1536Y primary antibody) in green, and glial fibrillary acidic protein (GFAP: astrocytes) in red. Scale bar: 50 μm. (TIF) [file ppat.1010956.s001.tif]

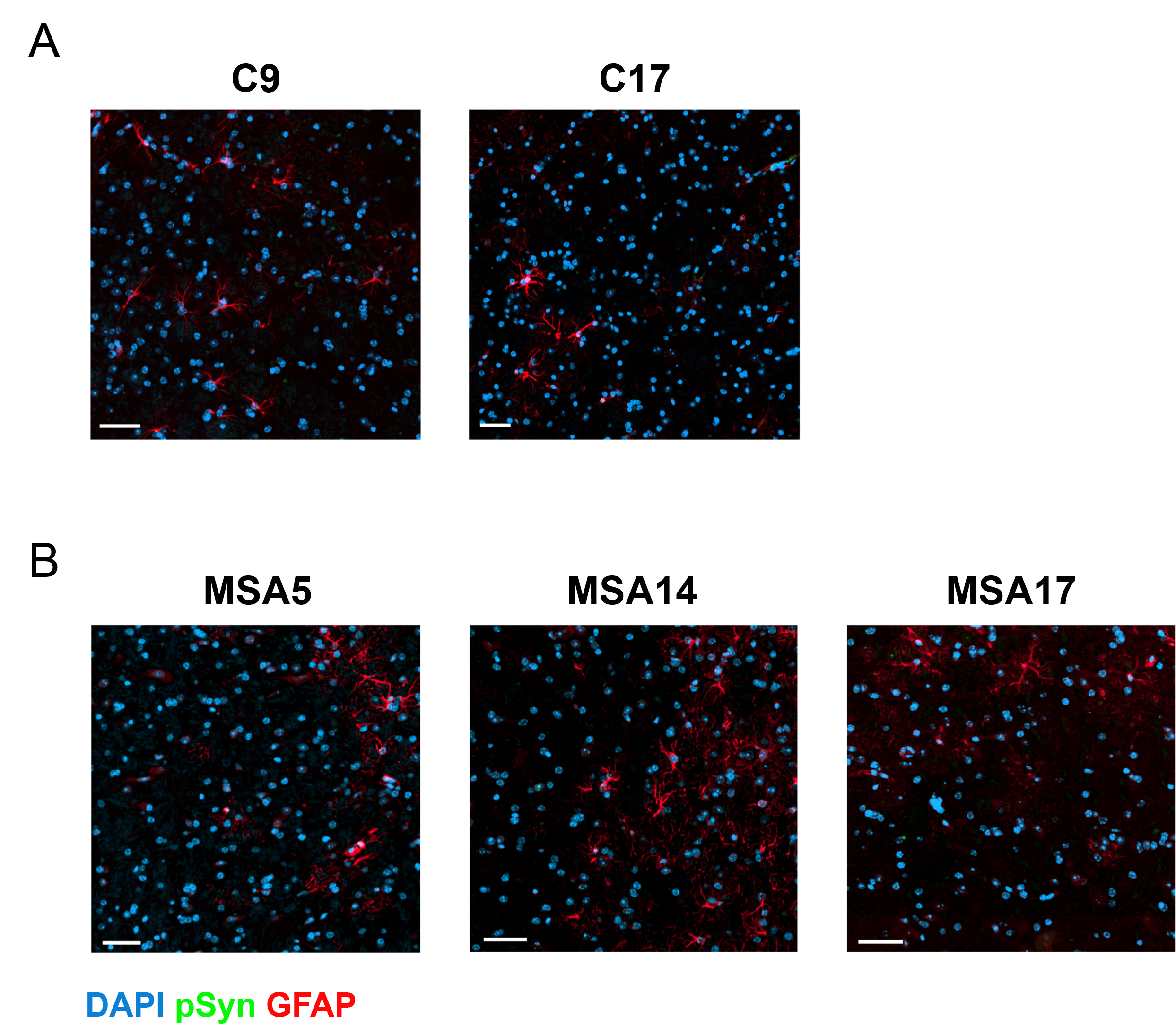

Supplement: S2 Fig — Fixed half-brains from TgM47+/- mice inoculated with either control (C9 or C17) or MSA (MSA5, MSA14, or MSA17) patient samples were collected 475 days postinoculation. Sections were immunostained using the EP1536Y (phosphorylated α-synuclein in green) and GFAP (red) primary antibodies. DAPI shown in blue. Neither the (A) control- nor the (B) MSA-inoculated mice developed α-synuclein inclusions. Scale bar: 50 μm. (TIF) [file ppat.1010956.s002.tif]

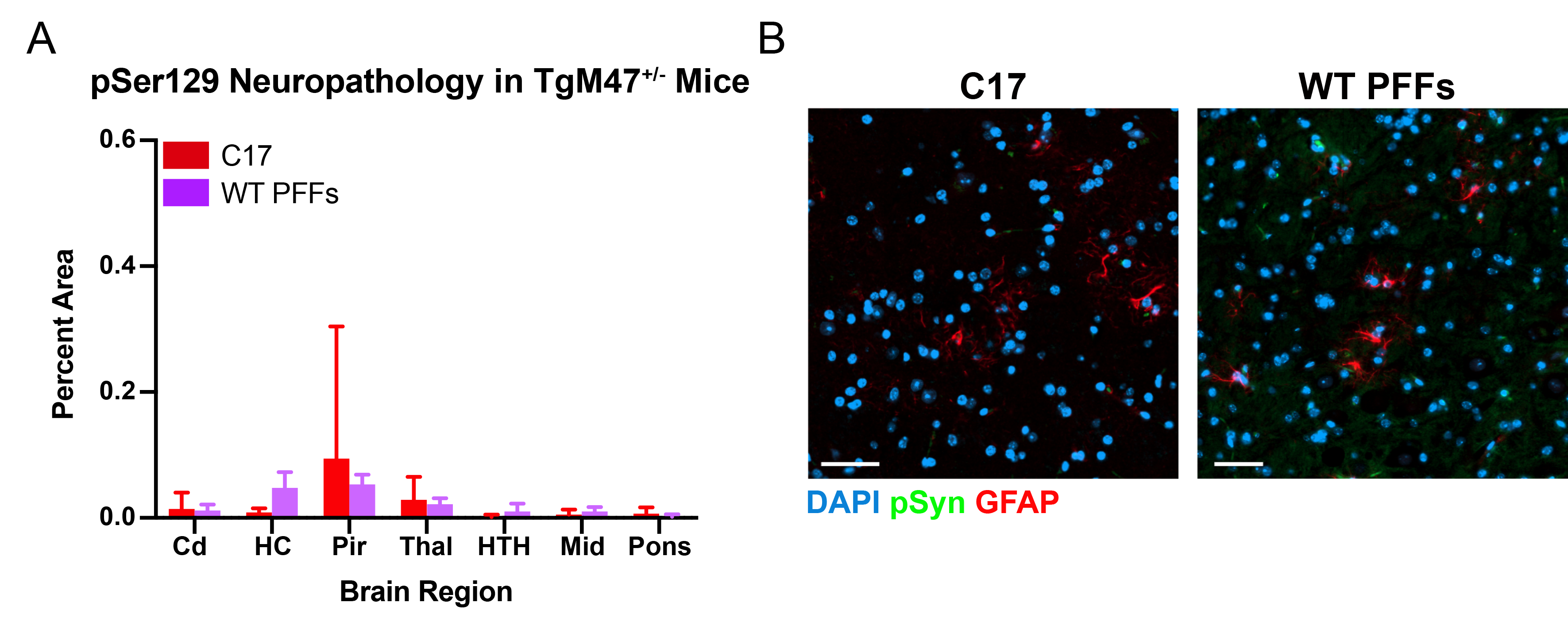

Supplement: S3 Fig — Fixed tissues from TgM47+/- mice inoculated with a control patient sample (C17) collected 475 days postinoculation (dpi) or WT PFFs collected 531 dpi were immunostained for phosphorylated α-synuclein (EP1536Y primary antibody) and GFAP. (A) Quantification of stained brain slices showed no phosphorylated α-synuclein inclusions were present in the caudate (Cd), hippocampus (HC), piriform cortex and amygdala (Pir), thalamus (Thal), hypothalamus (HTH), midbrain (Mid), or pons. (B) Representative images of the pons from mice inoculated with either control patient sample C17 (left) or WT PFFs (right). Phosphorylated α-synuclein in green, GFAP in red, and DAPI in blue. Scale bar: 50 μm. (TIF) [file ppat.1010956.s003.tif]
