# Supplementary material for: The E46K mutation modulates α-synuclein prion replication in transgenic mice
Source: PLoS Pathog. 2022 Dec 1;18(12):e1010956. doi: 10.1371/journal.ppat.1010956 (PMC9714912; doi:10.1371/journal.ppat.1010956)
Supplement: S1 Table — (DOCX) [file ppat.1010956.s004.docx]

S1 Table. Infectivity of aged TgM47^+/-^ samples in cultured cells.

| Mean cell infection ± SD  (Fluorescence/Cell × 10^3^ A.U.) | | | | |
| --- | --- | --- | --- | --- |
| α-syn140-YFP | α-syn140*E46K-YFP | α-syn140*A53T-YFP | α-syn140*E46K,A53T-YFP | α-syn95*A53T-YFP |
| 1.0 ± 0.5 | 0.8 ± 0.2 | 1.7 ± 0.8 | 0.8 ± 0.4 | 3.4 ± 0.1 |
