# Supplementary material for: The E46K mutation modulates α-synuclein prion replication in transgenic mice
Source: PLoS Pathog. 2022 Dec 1;18(12):e1010956. doi: 10.1371/journal.ppat.1010956 (PMC9714912; doi:10.1371/journal.ppat.1010956)
Supplement: S2 Table — (DOCX) [file ppat.1010956.s005.docx]

S2 Table. Patient sample information.

| Patient | Disease | Age at Death | Sex | Brain Region | Brain Bank |
| --- | --- | --- | --- | --- | --- |
| C9 | None | 62 | M | Putamen | University of Miami |
| C17 | None | 65 | M | Putamen | University of Miami |
| MSA5 | MSA | 60 | M | Basal ganglia | Parkinson’s UK |
| MSA14 | MSA | 76 | M | Basal ganglia | MADRC^a^ |
| MSA17 | MSA | 60 | M | Substantia nigra | MADRC^a^ |

^a^Massachusetts Alzheimer’s Disease Research Center
