# Supplementary material for: The E46K mutation modulates α-synuclein prion replication in transgenic mice
Source: PLoS Pathog. 2022 Dec 1;18(12):e1010956. doi: 10.1371/journal.ppat.1010956 (PMC9714912; doi:10.1371/journal.ppat.1010956)
Supplement: S1 Text — (DOCX) [file ppat.1010956.s006.docx]

# Supplementary Materials and Methods

## Human patient neuropathology

Neuropathology in human tissue samples received from the Parkinson’s UK Brain Bank was assessed following bisection of the brain (one hemisphere fixed in 10% buffered formalin and the other hemisphere sliced coronally, photographed on a grid, and rapidly frozen). Fixed tissue blocks from 20 key brain regions were stained with H&E and Luxol fast blue (LFB). To diagnose and stage disease, appropriate blocks were stained with antibodies against α-synuclein, β-amyloid, tau, and p62. An MSA diagnosis was based on α-synuclein inclusions in oligodendrocytes [1].

Patient samples obtained from the Massachusetts Alzheimer’s Disease Research Center (ADRC) Brain Bank were assessed to confirm the diagnosis of MSA. Fresh brains were dissected down the midline with one half fixed in 10% (vol/vol) neutral buffered formalin and coronally sectioned and the other half coronally sectioned before rapid freezing. The fixed tissue was evaluated histologically using a set of blocked regions representative of a variety of neurodegenerative diseases. All blocks were stained with LFB and H&E. Selected blocks were used for immunohistochemical staining for α-synuclein, β-amyloid, and phosphorylated tau. A confirmed MSA diagnosis required the presence of glial cytoplasmic inclusions [2].

**REFERENCES**

1. Alafuzoff I, Ince PG, Arzberger T, Al-Sarraj S, Bell J, Bodi I, et al. Staging/typing of Lewy body related alpha-synuclein pathology: a study of the BrainNet Europe Consortium. Acta Neuropathol. 2009;117:635–52.

2. Gilman S, Wenning GK, Low PA, Brooks DJ, Mathias CJ, Trojanowski JQ, et al. Second consensus statement on the diagnosis of multiple system atrophy. Neurology. 2008;71:670–6. PubMed Central PMCID: PMCPMC2676993.

3. Emmer KL, Waxman EA, Covy JP, Giasson BI. E46K human alpha-synuclein transgenic mice develop Lewy-like and tau pathology associated with age-dependent, detrimental motor impairment. J Biol Chem. 2011;286:35104–18.
